# The Clinical and Radiological Evaluation of Far Cortex Locking Plate in Distal Femur Fractures

**DOI:** 10.7759/cureus.14289

**Published:** 2021-04-04

**Authors:** Gur Aziz Singh Sidhu, Hakam Singh, Harpal Selhi, Neil Ashwood

**Affiliations:** 1 Trauma and Orthopaedics, University Hospitals of Derby and Burton NHS Foundation Trust, Burton-on-Trent, GBR; 2 Trauma and Orthopaedics, Dayanand Medical College and Hospital, Ludhiana, IND

**Keywords:** far cortex locking (fcl), locking plates, distal femur fractures, fracture healing

## Abstract

Introduction

Locking plates in distal femur fractures were associated with a high rate of non-union and hardware failure. To overcome these drawbacks far cortex locking (FCL) concept was introduced. It is a novel bridge plating strategy to enhance interfragmentary motion for the promotion of secondary bone healing while retaining sufficient construct strength. The present study evaluated the effects of diaphyseal FCL fixation on fracture healing for periarticular locking plates used for fixation of distal femur fractures.

Materials and methods

Our cohort was of 11 consecutive patients who presented to emergency after distal femur fracture and underwent surgery with the FCL plate between January 2015 and January 2016. Clinical (KOOS) and radiological evaluation of all patients was done to look for knee scores and union. Also, other complications like infection, non-union, painful hardware, implant failure were recorded

Results

No non-union or hardware failure was observed in our cohort of 11 patients. Early callus formation was seen and partial weight-bearing was started at an average of 6 weeks (5-8 weeks). Average time to clinical healing was 10 weeks (8-13 weeks) whereas radiographic union was seen at 16 weeks (14-17 weeks). One patient with an open fracture had superficial surgical wound infection which healed uneventfully after one debridement and with IV antibiotics. The average knee injury and osteoarthritis outcome score (KOOS) at final follow-up was 91 (87-95) in our cohort.

Conclusion

FCL is an effective method to reduce construct stiffness, promote early callus formation, decrease non-union rate and achieve biological healing while retaining sufficient strength to prevent hardware failure.

## Introduction

Plate osteosynthesis has undergone a substantial evolution over few decades [1-4]. Locked plating was believed to represent a solution for many issues associated with compression plating [4]. Similar to standard plating, locked plating has its own problems and pitfalls [5]. The occurrence of nonunion at fracture site with stable and rigid fixation has been puzzling [5]. It is has been documented that motion at the fracture site is essential for secondary bone healing [4-6] However, the only drawback for fracture healing is the rigidity provided by locking screws. A stiff implant reduces micromotion at the fracture site, thus leading to nonunion and subsequent implant failure [5,6].

Several strategies for reducing the stiffness of locked plating have been studied [1-6]. These include decreasing the plate thickness, increasing the plate elevation, and increasing the plate span. While these strategies are effective for reducing the stiffness of locked plating, they also reduce their strength. To overcome the drawbacks of locked plating, a new strategy, termed far cortex locking (FCL) has been tested [5-8] Far cortical locking may provide a novel bridge plating strategy to enhance interfragmentary motion for the promotion of secondary bone healing while retaining sufficient construct strength [5,7,8].

The present study evaluated the effects of diaphyseal FCL fixation on fracture healing for periarticular locking plates used for fixation of distal femur fractures. Results of the present study tested the hypothesis that diaphyseal fixation of a periarticular femur plate with FCL screws will reduce construct stiffness and will induce early callus formation and union and will reduce complications like nonunion, implant breakage without decreasing construct strength compared to plate application using standard locking screws.

## Materials and methods

It was a prospective study of 11 consecutive patients who presented to emergency after distal femur fracture and underwent surgery with the FCL plate between January 2015 and January 2016. The patients were evaluated in the accident and emergency (A/E) and managed according to the standard Advanced trauma life support protocol. The patients with pathological fractures, polytrauma patients, blunt trauma chest or abdomen and concomitant tibia or acetabular fractures or non-unions were excluded from the study.

The mean age of patients was 46.2 years (range 14-76 years). Roadside accidents were the cause of injury in eight cases whereas three patients had domestic falls. Five fractures were AO/OTA Type 33A while six patients suffered Type 33C fractures. There was one patient with open fracture, whereas the rest had closed fractures. One patient with open fracture was managed with spanning external fixator across knee and then definite fixation was done after 12 days. 

All surgeries were performed by a senior surgeon under regional anesthesia. Tourniquet was used in all cases. Distal femur FCL plate pre-contoured for the lateral femoral condyle was used and at least three 5-mm FCL screws were inserted in the shaft and regular locking screws in the femur condyles. A locking cap screw was applied to lock the screw head to the plate to generate an angle-stable construct after the insertion of each screw. Postoperative, the knee was protected with knee immobilizer. Physiotherapy in form of knee mobilization was started once the pain subsides or patient was comfortable. Suture was removed on the 12th post-operative day. The patients were regularly followed up at 6 weeks, 12 weeks, 6 months and 12 months. The clinical and radiological data were recorded for all patients.

## Results

**Figure 1:**
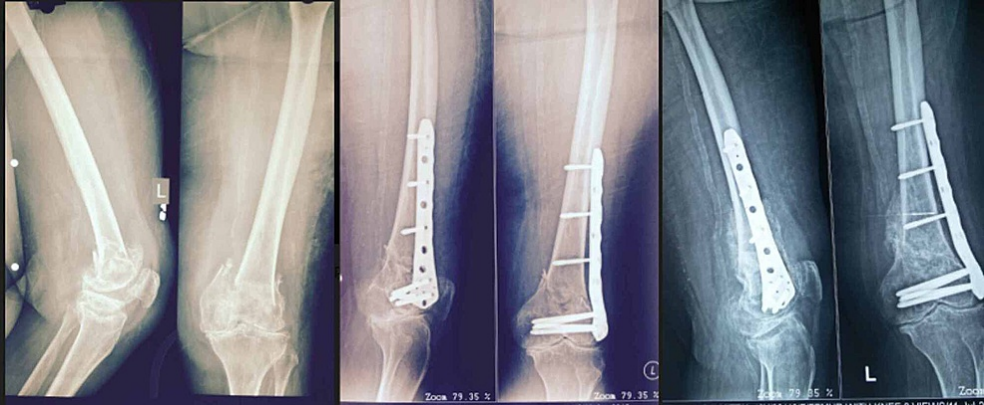
Preoperative, two weeks and six-month follow-up radiographs.

No non-union or hardware failure was observed in our cohort of 11 patients. Early callus formation was seen and partial weight-bearing was started at an average of 6 weeks (5-8 weeks). Average time to clinical healing was 10 weeks (8-13 weeks) whereas radiographic union was seen at 16 weeks (14-17 weeks) (Figure 1).

**Figure 2:**
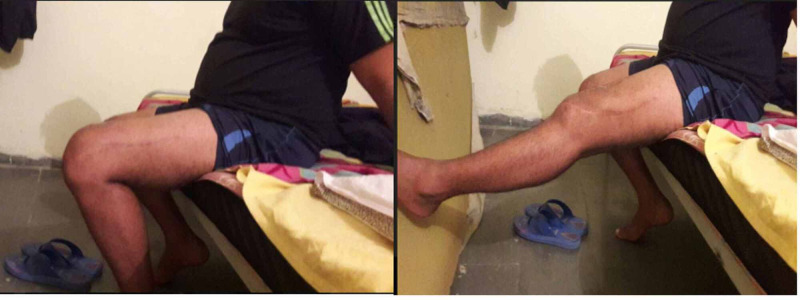
Post-operative range of motion at knee.

One patient with open fracture had superficial surgical wound infection which healed uneventfully after one debridement and with IV antibiotics. Range of motion exercises were started on day 2 and four of the 11 patients were having knee flexion greater than 90 degrees at discharge. At the final follow-up, 10 of the 11 patients had average range of motion of 95 degrees (90-105 degrees) (Figure 2). The average knee injury and osteoarthritis outcome score (KOOS) at final follow-up was 91 (87-95) in our cohort.

## Discussion

The far cortical locking system promotes secondary bone healing by reducing stiffness of the construct, nearly parallel interfragmentary motion and biphasic stiffness [9]. Moreover, some studies have shown that FCL constructs led to formation of more callus, stronger healing and prevented non-unions associated with locking plates [10].

Doornink and co-workers studied standard locking plate and FCL for 22 paired femurs for stiffness and durability under dynamic loading [7]. They reported that FCL group had 81% lower initial stiffness. Also under body weight loading, this group exhibited five times more interfragmentary motion than locking plate group (p < 0.001). They concluded that FCL fixation led to a reduction in stiffness thus promoted fracture healing through secondary intention [7].

In vivo study, Bottlang and colleagues demonstrated the effect of FCL on fracture healing using ovine tibial osteotomy model [6]. They used standard locked plating and FCL constructs to stabilize tibial osteotomies with a gap of 3 mm. They found that FCL plates had 84% lower initial stiffness and provided parallel interfragmentary motion. Moreover, the FCL constructs demonstrated significantly more callus on weekly radiographs (p = 0.004). The callus formed was symmetric in FCL group as compared to asymmetric in standard locking group. Additionally, histological sections depicted `partial non-unions' in 50% of LP constructs whereas the FCL group showed bridging callus over near and far cortices [6]. Our results of early healing and bridging callus formation had been consistent with their results.

In this retrospective case series by Adams and colleagues, 15 patients (two with bone loss) with a distal femur fracture treated with FCL system were analyzed [8]. They reported no case of nonunion or implant failure and the average time to union was 24 weeks. All fractures, including the two with bone loss, healed without intervention. There was one reoperation due to hardware problems. They concluded that far cortical locking screws may be a solution to high nonunion rate associated with distal femur fractures treated with traditional locked constructs. Our results with FCL system were similar, although our cohort was smaller. However, the results are promising and could instigate further interest as well as research into FCL idea [8].

Henderson et al. retrospectively studied 82 patients treated with 86 distal femur fractures using lateral locking plates [11]. The reported 20 % of fractures failed to unite, less callus formed in fractures with non-unions in patients treated with stainless steel plates. Moreover, 28 of 70 fractures had complications and 19 of which had another surgery. They concluded callus inhibition as primary cause for such a high rate of nonunion in distal femur fractures fixed using locking plates. We did not have any non-union in our cohort thus supporting the fact that FCL system leads to interfragmentary micromotion and fracture healing by secondary intention [11].

Our study had few limitations. Small number of cases in our cohort and the absence of comparison group were amongst them. However, the results were encouraging and could lead to further research on the topic and further studies are needed. 

## Conclusions

FCL is an effective method to reduce construct stiffness, promote early callus formation, decrease non-union rate and achieve biological healing while retaining sufficient strength to prevent hardware failure.
